# The Role of the Organization of Light-Harvesting Complex II in the Drought Sensitivity of *Pisum sativum* L.

**DOI:** 10.3390/ijms262211078

**Published:** 2025-11-16

**Authors:** Georgi D. Rashkov, Martin A. Stefanov, Preslava B. Borisova, Anelia G. Dobrikova, Emilia L. Apostolova

**Affiliations:** Institute of Biophysics and Biomedical Engineering, Bulgarian Academy of Sciences, Acad. G. Bonchev Str., Bl. 21, 1113 Sofia, Bulgaria; megajorko@abv.bg (G.D.R.); martin_12.1989@abv.bg (M.A.S.); pborisova@bio21.bas.bg (P.B.B.); aneli@bio21.bas.bg (A.G.D.)

**Keywords:** pea mutants, PEG-induced drought stress, photosynthetic apparatus, pigment content, PAM chlorophyll a fluorescence, JIP test, water deficit

## Abstract

Drought stress is a major abiotic factor limiting plant growth and productivity. This study investigates the role of oligomerization of the light-harvesting complex of photosystem II (LHCII) in modulating plant responses to drought stress. Using pea plants (*Pisum sativum* L.): Borec (wild type) and its mutants *Costata* 2/133 and *Coeruleovireus* 2/16, with different degrees of LHCII oligomerization, we examined the impact of water deficit on the functions of the photosynthetic apparatus. This study demonstrated that plants with a higher degree of LHCII oligomerization (wild type and *Coeruleovireus* 2/16) have enhanced drought tolerance, expressed by reduced lipid peroxidation and membrane damage, protection of the photosynthetic pigment content, which corresponds with better photosynthetic performance. Data revealed only minor drought-induced inhibition of photosystem II (PSII) photochemistry (Fv/Fm, Φ_PSII_), electron transport rate (ETR), and rate of photosynthesis (R_Fd_)), along with sustained performance indices (PI_ABS_ and PItotal) in plants with higher LHCII oligomerization compared to those with lower levels (*Costata* 2/133). Additionally, the current study indicates that under drought stress and low actinic light, the interaction with plastoquinone and controlled dissipation of excess energy are promoted in thylakoid membranes with increased LHCII oligomerization. In contrast, drought-stressed plants with lower oligomerization (*Costata* 2/133) showed a significant increase in non-regulated energy losses under high actinic light. These results highlight the protective function of LHCII oligomerization in preserving photosynthetic integrity and functioning under drought stress and suggest that it could be a promising target for enhancing crop resilience in a changing climate.

## 1. Introduction

Plants are exposed to a wide range of environmental stresses during their growth and development. These stresses are broadly categorized into biotic stresses (caused by pathogens and insects) and abiotic stresses such as drought, salinity, extreme temperatures, and light intensity. Among these, drought stress is considered one of the most destructive abiotic factors affecting plant health and productivity [1,2,3], influencing plant physiology by destabilizing all essential processes [4,5,6,7]. However, the impact of drought stress on plants depends on several factors, including soil moisture gradient, light intensity, temperature, plant species, and developmental stage [4,5,8]. In plants sensitive to drought, genes encoding important regulatory enzymes involved in the light and dark reactions of photosynthesis are significantly downregulated [6]. Drought stress influences plant water relations and reduces water-use efficiency, which leads to changes in plant morphology—from chloroplast architecture and protein conformational dynamics to the functional modulation of photosynthetic complexes [5,9,10,11]. Previous studies have revealed that drought induces changes in the thylakoid membranes, associated with a decrease in the number of layers and grana [11,12]. 

Oxidative stress caused by drought has a significant impact on plants. Increased levels of reactive oxygen species (ROS), triggered by a disruption in the balance between their production and detoxification, can damage DNA, pigments, proteins, and other essential molecules [13,14], causing changes in the organization of complexes of thylakoid membranes [15,16]. The antioxidant systems of plants, including both enzymatic and non-enzymatic components, protect them from the enhanced accumulation of ROS [17,18,19,20]. Under abiotic stress, plants undergo various biochemical and anatomical changes to cope with these adverse conditions, including stomatal closure, altered root growth and architecture, changes in metabolic pathways, and altered physiological responses. Furthermore, water deficit has a detrimental effect on nutrient absorption, membrane permeability, and chlorophyll synthesis, thereby diminishing photosynthetic efficiency, which lowers plant growth and yields [21].

Multiple transcription factor (TF) families play essential roles in regulating plant metabolism under abiotic stress conditions, including drought, salinity, and cold stress [22]. Among them, the WRKY, MYB, and NAC families have been widely recognized for their involvement in stress-responsive gene expression [23,24,25,26]. For example, MbWRKY50, a WRKY transcription factor from *Malus baccata*, has been shown to enhance drought and cold tolerance by upregulating antioxidant capacity and reducing oxidative damage [23]. Similarly, MbICE1, an inducer of CBF expression, plays a central role in cold and drought resistance. Overexpression of MbICE1 in *Arabidopsis thaliana* led to increased chlorophyll and proline content, while reducing the accumulation of malondialdehyde (MDA), hydrogen peroxide (H_2_O_2_), and superoxide anion (O_2_^−^) [24]. In grapevine (*Vitis heyneana*), VhMYB2 and VhWRKY44 have been implicated in enhancing salinity and drought tolerance through regulation of osmotic balance and antioxidant defense mechanisms [25,27]. Furthermore, in *Fragaria vesca*, FvMYB44 and FvNAC29 have been identified as key regulators of drought-responsive pathways, contributing to improved stress resilience [26,28]. These findings underscore the importance of transcriptional regulation in plant adaptation to abiotic stress and highlight potential targets for improving crop tolerance.

A major impact of drought stress is the inhibition of photosynthesis, which varies depending on the duration and severity of water deficiency, as well as the plant species [4,29,30]. Drought-induced changes in the organization of the pigment–protein complexes [11] and in the lipids of thylakoid membranes [31] inhibit the light reactions of photosynthesis, as the primary target is PSII photochemistry [4,32,33]. This has been shown to influence photosynthetic efficiency by compromising electron-transport chains, photochemical quenching mechanisms, and the regulation of excess energy dissipation [4,34,35,36]. Previous studies also revealed that water deficiency decreases the maximum efficiency of PSII photochemistry (Fv/Fm), the amount of open PSII reaction centers, and the electron transport rate (ETR), while increasing the non-photochemical quenching [37,38]. A factor influencing the function of the photosynthetic apparatus is the reduction in chlorophyll content under water deficit, which affects the light-harvesting ability [39]. Changes in chlorophyll content vary depending on the drought tolerance of the plants [40]. These changes in the function of the photosynthetic apparatus correspond with a reorganization of the PSII complex [41,42]. Under short-term drought stress, the light-harvesting complex of PSII (LHCII) is detached from the PSII core complex, while after long-term drought stress, most photosynthetic proteins are decreased [37,41]. Immunoblotting analysis of PSII proteins from pea plants revealed enhanced degradation of CP43 and D1 proteins under water deficiency [43]. At the same time, major PSII antenna proteins and PSII core proteins are downregulated [44]. The decreased content of the light-harvesting complexes under drought corresponds to downregulated chlorophyll (Chl) biosynthesis [32]. Under prolonged drought stress, the structural and functional integrity of the photosynthetic machinery begins to be disrupted [43]. It has been shown that the destabilization of the oxygen-evolving complex (OEC) under drought is accompanied by degradation of the manganese-stabilizing protein (PsbO) [29]. Although more stable than D1, the D2 protein also undergoes cofactor destabilization and reduced synthesis, further diminishing the efficiency of the PSII reaction center [43]. At a later stage, under prolonged drought stress, the PSI complex is disassembled, and the light-harvesting antenna of PSI is reduced [11,41,45]. 

In nature, drought stress is often combined with high-light stress. On sunny days, plants absorb significantly more light over several hours than they can effectively utilize due to daily variations in irradiation. A study with pea plants has shown that a reorganization of PSII core components, notably D1 and CP43, occurs alongside elevated photoinhibition. Moreover, structural destabilization of grana in thylakoid membranes has been observed [43,46,47,48]. 

Pea (*Pisum sativum* L.) is one of the most important legumes cultivated worldwide, but its growth and productivity are strongly influenced by environmental factors [49]. As a member of the Fabaceae family, the pea is highly sensitive to drought stress [50]. Water deficit adversely affects pea plants by reducing growth, yield, pigment concentration, and photosynthetic efficiency, with the severity of impact varying across different pea varieties [51,52]. Previous studies have reported a decline in both maximum and operating quantum efficiency of PSII photochemistry, along with a significant reduction in CO_2_ assimilation due to substantial stomatal closure [53]. Additionally, osmolytes have been shown to play a crucial role in the adaptation of pea plants to water scarcity [54]. 

Investigations with pigment pea mutants demonstrated a relationship between the organization of PSII and the sensitivity of the photosynthetic apparatus to abiotic stress (high light, UV-A radiation, high temperature) [55,56,57]. The different tolerance of plant species to stress factors, as well as the strong effect of drought on the organization and function of the PSII complex (LHCII–PSII core), raises the question of the influence of LHCII organization on drought tolerance in plants. We hypothesize that an increase in the degree of LHCII oligomerization (the ratio of oligomeric to monomeric forms of LHCII, LHCIIo/LHCIIm) is related to drought tolerance of plants. In the present study, pea plants (*Pisum sativum* L.): Borec (wild type) and its mutants *Costata* 2/133 and *Coeruleovireus* 2/16, with different degrees of LHCII oligomerization, were used. The studied plants are characterized by variations in pigment content, structural organization, surface electric properties, and the ratios of LHCII/PSII, as well as the LHCIIo/LHCIIm ratio [58,59]. In our previous studies, we showed that the LHCIIo/LHCIIm ratio increased in the following order: *Costata* 2/133 < wild type < *Coeruleovireus* 2/16 [58,59]. This increase was accompanied by a decrease in transversal charge asymmetry and the net electric charge of thylakoid membranes [58]. Differences in the organization of LHCII–PSII in the studied plants influenced energy distribution between the two photosystems, affected excitation energy transfer from chlorophyll b to chlorophyll a, and affected the kinetic parameters of oxygen-evolving activity [59]. A recent study revealed that LHCII organization plays a key role in the functional efficiency of the photosynthetic apparatus [60]. Therefore, this study aimed to investigate, for the first time, the sensitivity of pea plants with different LHCII organization to drought stress.

## 2. Results

### 2.1. Stress Markers

**Figure 1:**
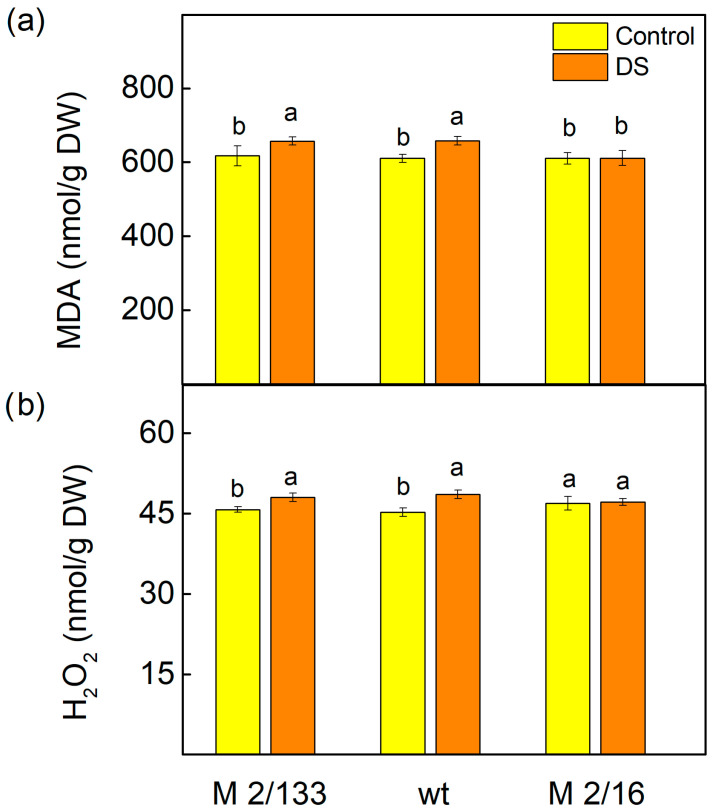
The amounts of malonaldehyde (MDA) (**a**) and hydrogen peroxide (H_2_O_2_) (**b**) in Borec wild type (wt) and its mutants *Costata* 2/133 (M 2/133) and *Coeruleovireus* 2/16 (M 2/16) in plants untreated (control) and treated with 20% PEG (DS). Different letters indicate significant differences among variants at *p* < 0.05.

The data showed that drought stress (20% PEG) caused only a smaller increase in stress markers (H_2_O_2_ and MDA) in both wild type (wt) and mutant *Costata* 2/133 (M 2/133) plants, relative to their respective controls (Figure 1). In these plants, characterized by a lower LHCIIo/LHCIIm ratio, H_2_O_2_ and MDA levels rose by just 6% to 9%. In contrast, the mutant *Coeruleovireus* 2/16 (M 2/16), which exhibits enhanced LHCII oligomerization, maintained H_2_O_2_ and MDA concentrations similar to those observed in untreated plants. 

### 2.2. Pigment Composition

**Figure 2:**
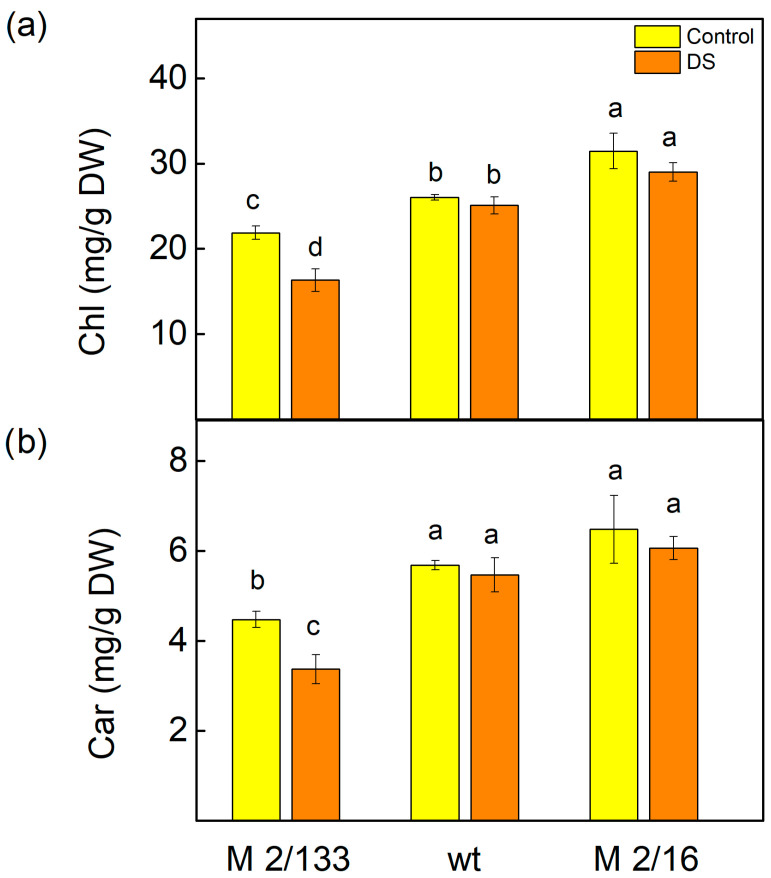
The amounts of total chlorophylls (Chl) (**a**) and carotenoids (Car) (**b**) in leaves of Borec wild type (wt) and its mutants *Costata* 2/133 (M 2/133) and *Coeruleovireus* 2/16 (M 2/16) in plants untreated (control) and treated with 20% PEG (DS). Different letters indicate significant differences among variants at *p* < 0.05.

Analysis of leaf pigments showed that treatment with 20% PEG significantly affected total chlorophyll (Chl) levels (Figure 2). In M 2/133, Chl content decreased by 25%, whereas in wt and M 2/16, levels remained comparable to untreated plants, with no statistically significant differences (*p* < 0.05). Similarly, carotenoid (Car) content declined by 25% in M 2/133 after drought treatment, while changes in wt and M 2/16 were not statistically significant (*p* < 0.05) relative to their respective controls. Additionally, both treated and untreated wt and M 2/16 plants exhibited higher Chl and Car levels than M 2/133 (Figure 2). 

### 2.3. Membrane Stability Index and Relative Water Content

**Figure 3:**
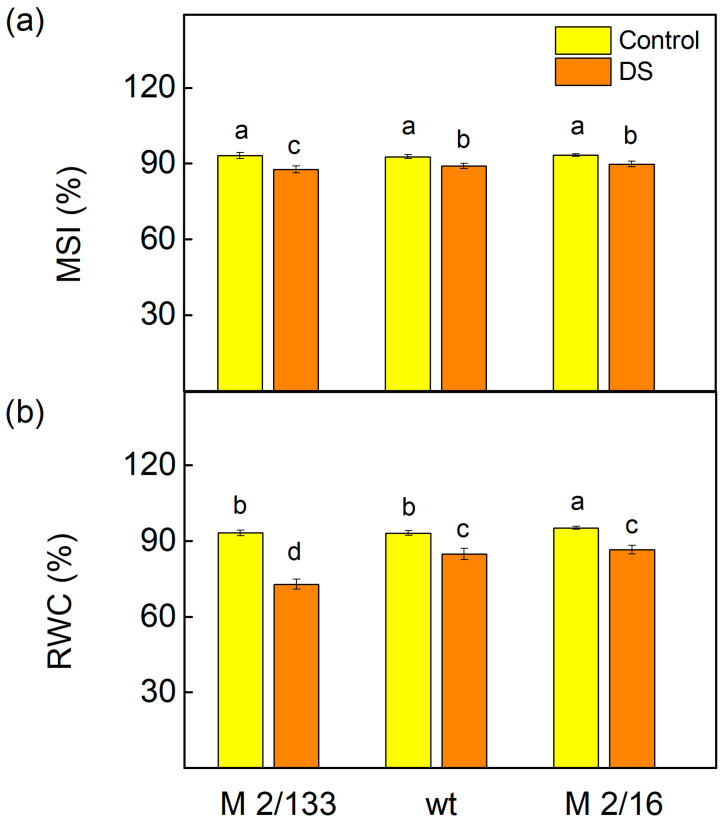
The membrane stability index, MSI (**a**) and relative water content, RWC (**b**) of Borec wild type (wt) and its mutants *Costata* 2/133 (M 2/133) and *Coeruleovireus* 2/16 (M 2/16) in plants untreated (control) and treated with 20% PEG (DS). Different letters indicate significant differences among variants at *p* < 0.05.

Membrane injury in the leaves of the studied pea plants under drought stress was assessed using the membrane stability index (MSI), a rapid marker for the determination of membrane integrity under water deficit conditions [61]. Data revealed that the decrease in MSI is stronger in M 2/133 than in wt and M 2/16 (Figure 3). Furthermore, under water-deficient conditions, a greater reduction in leaf relative water content (RWC) was again observed in M 2/133 (by 20%) compared to the wt and M 2/16 (Figure 3). 

### 2.4. Pulse Amplitude Modulated Chlorophyll a Fluorescence 

**Figure 4:**
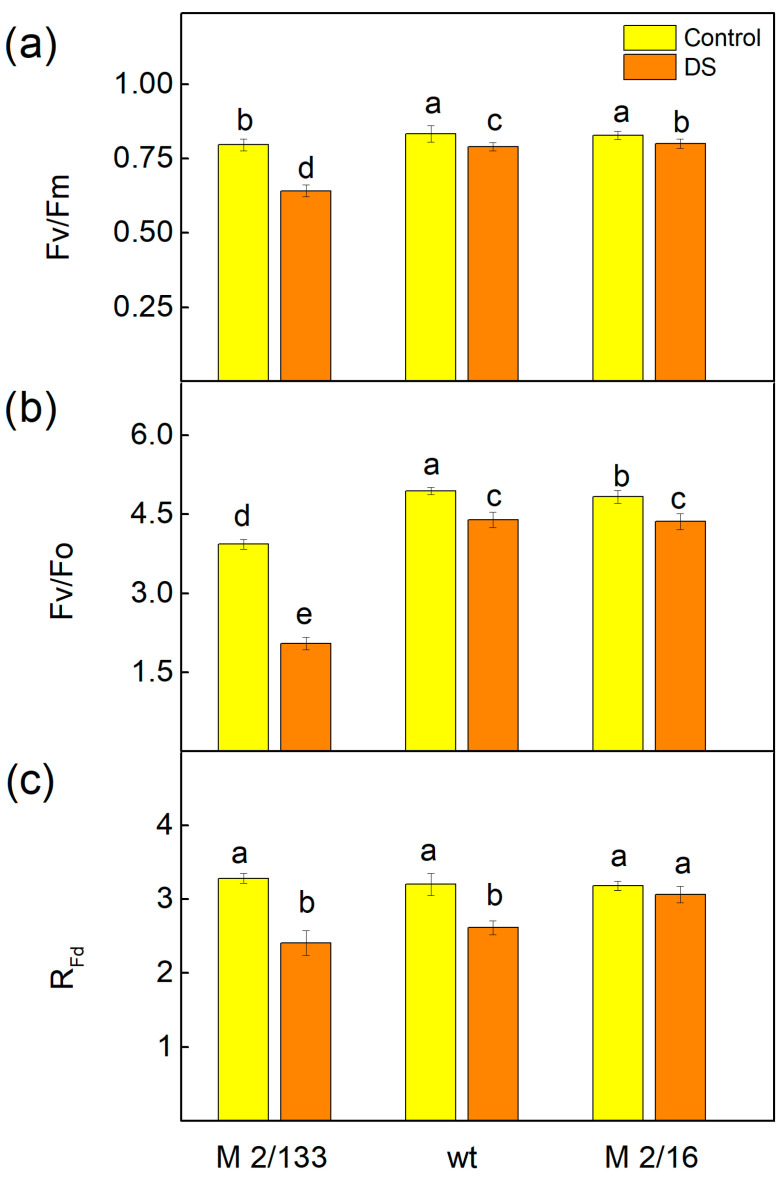
Selected PAM parameters of Borec wild type (wt) and its mutants *Costata* 2/133 (M 2/133) and *Coeruleovireus* 2/16 (M 2/16) in plants untreated (control) and treated with 20% PEG (DS). (**a**) Fv/Fm—the maximal quantum yield in dark-adapted state; (**b**) Fv/Fo—the ratio of the intensity of chlorophyll a fluorescence caused by photochemical processes to the intensity of the chlorophyll *a* fluorescence not excitonically bound to the reaction centers of PSII; (**c**) R_Fd_—chlorophyll fluorescence decay ratio. Different letters indicate significant differences among variants at *p* < 0.05.

Analysis of the Pulse Amplitude Modulated (PAM) chlorophyll a fluorescence signals showed that the maximal quantum yield in the dark-adapted state (Fv/Fm), and the ratio of chlorophyll a fluorescence intensity caused by photochemical processes to that not excitonically bound to PSII reaction centers (Fv/Fo), were less affected by drought stress in plants with higher LHCII oligomerization (wt and M 2/16) (Figure 4). The decrease in Fv/Fm was 6% in wt and 4% in M 2/16, while in M 2/133 it reached 20%. The data also revealed a stronger reduction in Fv/Fo in M 2/133 (by 46 %), compared to 10% in wt and M 2/16 (Figure 4). Furthermore, the parameter R_Fd_, which correlates with the rate of photosynthesis [62], decreased by 25% in M 2/133 and by 19% in wt, while in M 2/16 it remained unchanged (Figure 4).

**Figure 5:**
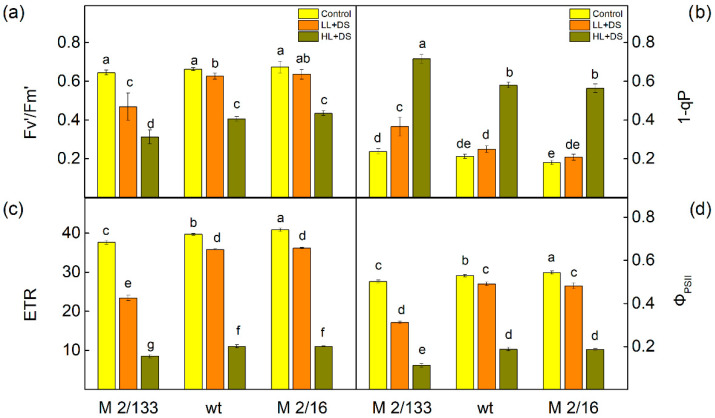
Selected PAM parameters of Borec wild type (wt) and its mutants *Costata* 2/133 (M 2/133) and *Coeruleovireus* 2/16 (M 2/16) in plants untreated (control) and treated with 20% PEG (DS). Plants were measured at low (LL, 150 µmoles photons/m^2^·s) and high (HL, 500 µmoles photons/m^2^·s) actinic light. (**a**) Fv′/Fm′—the effective quantum yield of PSII photochemistry; (**b**) 1-qP—the amount of the closed PSII reaction centers; (**c**) ETR—the electron transport rate; (**d**) Φ_PSII_—the effective quantum yield of energy conversion in PSII. Different letters indicate significant differences among variants for respective parameters at *p* < 0.05.

Experimental data indicated that drought stress induced variations in selected PAM parameters, influenced by the degree of LHCII oligomerization in thylakoid membranes and the intensity of actinic light: low light (LL, 150 µmol photons/m^2^·s) and high light (HL, 500 µmol photons/m^2^·s) (Figure 5). The impact of drought on Fv′/Fm′, 1-qP, ETR, and Φ_PSII_ was more pronounced in M 2/133 than in wt and M 2/16 under both light conditions. In M 2/133, the effective quantum yield of PSII photochemistry (Fv′/Fm′) decreased by 27% under LL and by 52% under HL. Concurrently, the proportion of closed PSII centers (1-qP) increased by 54% under LL and tripled under HL (Figure 5). The electron transport rate (ETR) was inhibited by 38% under LL in M 2/133, while wt and M 2/16 showed only a ~10% reduction. Under HL, ETR declined by 72–78% across all studied plants. The effective quantum yield of energy conversion in PSII (Φ_PSII_) was also more severely affected in M 2/133, decreasing by 38% under LL compared to a 10% reduction in wt and M 2/16. Under HL, Φ_PSII_ dropped by 78% in M 2/133 and by 65% in wt and M 2/16 (Figure 5).

**Figure 6:**
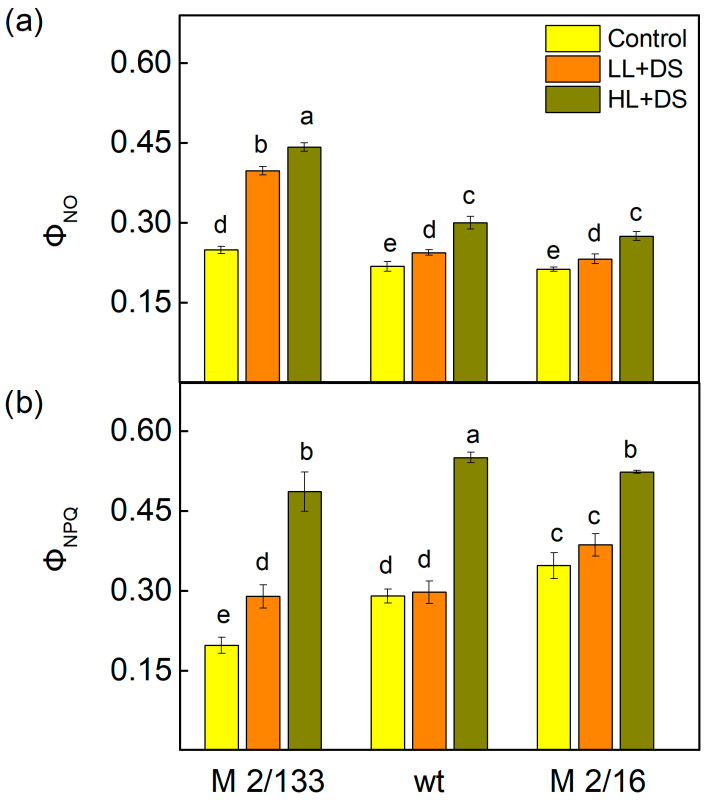
The non-regulated (Φ_NO_) (**a**) and regulated (Φ_NPQ_) (**b**) energy losses in Borec wild type (wt) and its mutants *Costata* 2/133 (M 2/133) and *Coeruleovireus* 2/16 (M 2/16) in plants untreated (control) and treated with 20% PEG (DS). Different letters indicate significant differences among variants for respective parameters at *p* < 0.05.

At the same time, an increase in the non-regulated (Φ_NO_) and regulated energy losses (Φ_NPQ_) in the plants after drought stress under both LL and HL actinic light was observed (Figure 6). The increase in Φ_NO_ was by 59% under LL and by 77% under HL in M 2/133, while in wt and M 2/16 it was by 9–12% under LL and by 29–38% under HL. The data also showed that under drought stress, the increase in Φ_NPQ_ was smaller than Φ_NO_ under LL conditions, while under HL, the increase in Φ_NPQ_ was bigger in comparison to that of Φ_NO_ (Figure 6). The regulated energy losses (Φ_NPQ_) increased by 46% under LL and by 146% under HL in M 2/133 compared to control plants. At the same time, the increase in Φ_NPQ_ was to a smaller extent in wt and M 2/16 than in M 2/133 (Figure 6).

**Figure 7:**
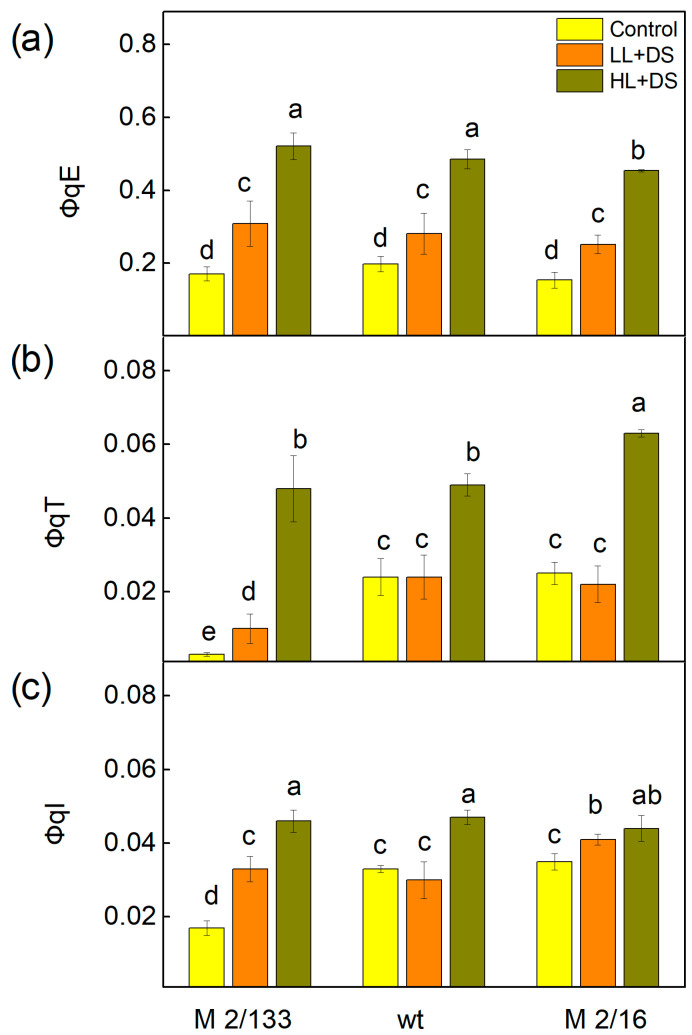
The quantum yields for different processes of the non-photochemical quenching in Borec wild type (wt) and its mutants *Costata* 2/133 (M 2/133) and *Coeruleovireus* 2/16 (M 2/16) at low (LL, 150 µmoles photons/m^2^·s) and high (HL, 500 µmoles photons/m^2^·s) actinic light in the control untreated plants and plants treated with 20% PEG (DS). (**a**) the quantum yield of the energy-dependent quenching—ΦqE; (**b**) the quantum yield of the state transition quenching—ΦqT; (**c**) the quantum yield of the photoinhibitory quenching—ΦqI. Different letters indicate significant differences among variants for respective parameters at *p* < 0.05.

The data in Figure 7 showed the influence of drought stress on the quantum yields of different non-photochemical quenching processes in Borec wild type (wt) and its mutants *Costata* 2/133 (M 2/133) and *Coeruleovireus* 2/16 (M 2/16). The quantum yield of the energy-dependent quenching (ΦqE) increased after treatment with 20% PEG, with the most pronounced effect observed in M 2/133 (by 81%) (Figure 7a). The data also showed that for this mutant plant (M 2/133), the value of the component ΦqE under HL was three times higher than in untreated plants. The quantum yield of the state transition quenching (ΦqT) increased only in M 2/133, while in wt and M 2/16, its values were similar to those of the control plants (Figure 7b). A strong increase in ΦqT was registered in plants treated with PEG under HL conditions. The quantum yield of photoinhibitory quenching (ΦqI) increased after drought stress only in M 2/133, while under HL conditions, its values rose in all drought-stressed plants compared to respective controls (Figure 7). This increase was again more pronounced in M 2/133 (by 170%). In plants with a higher degree of oligomerization, the component ΦqI was higher by 42% and by 26% in wt and M 2/16, respectively (Figure 7c).

Kinetics of the dark reduction in the chlorophyll *a* fluorescence after a saturated light pulse provides information about Q_A_^−^ reoxidation [63,64]. The data revealed that drought stress led to an increase in time t_1_, characterizing the interaction with plastoquinone (PQ). The increase was 27% in M 2/133, while in wt and M 2/16 it was 13% (Table 1). Time t_2_, characterizing the interaction of Q_A_ with OEC, increased in all studied plants. The parameter A_2_/A_1_ displays the interaction of Q_A_ with OEC (component A_2_) and the interaction with PQ (component A_1_). The value of this ratio was higher in M 2/133 than in wt and M 2/16 in both control and treated plants (Table 1).

### 2.5. Fast Chlorophyll a Fluorescence

**Figure 8:**
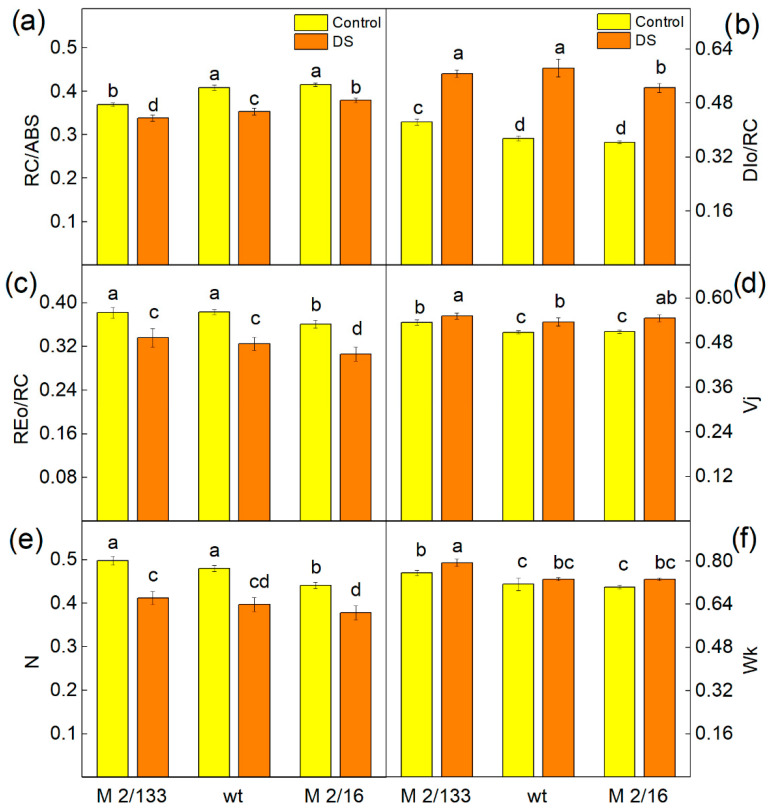
Selected JIP parameters: (**a**) RC/ABS—the reaction centers per PSII antenna chlorophyll; (**b**) Dio/RC—the light energy dissipation; (**c**) REo/RC—electron flux reducing end electron acceptors at the PSI acceptor side per reaction center; (**d**) Vj—the relative variable fluorescence at J-step; (**e**) N—maximum turnover of Q_A_ reducing until Fm was reached; (**f**) Wk—the ratio of the K phase to J phase measured in Borec wild type (wt) and its mutants *Costata* 2/133 (M 2/133) and *Coeruleovireus* 2/16 (M 2/16) in plants untreated (control) and treated with 20% PEG (DS). Different letters indicate significant differences among variants for respective parameters at *p* < 0.05.

Selected JIP parameters were used to characterize the functions of the photosynthetic apparatus after drought stress. Experimental results revealed that the reaction centers per PSII antenna chlorophyll (RC/ABS), maximum turnover of QA reducing until Fm was reached (N), and electron flux reducing end electron acceptors at the PSI acceptor side (REo/RC) decreased in all studied plants (Figure 7). The data also revealed an increase in the light energy dissipation (DIo/RC) and parameters Wk and Vj, characterizing the stability of the OEC and changes in the PSII acceptor side, respectively (Figure 8).

**Figure 9:**
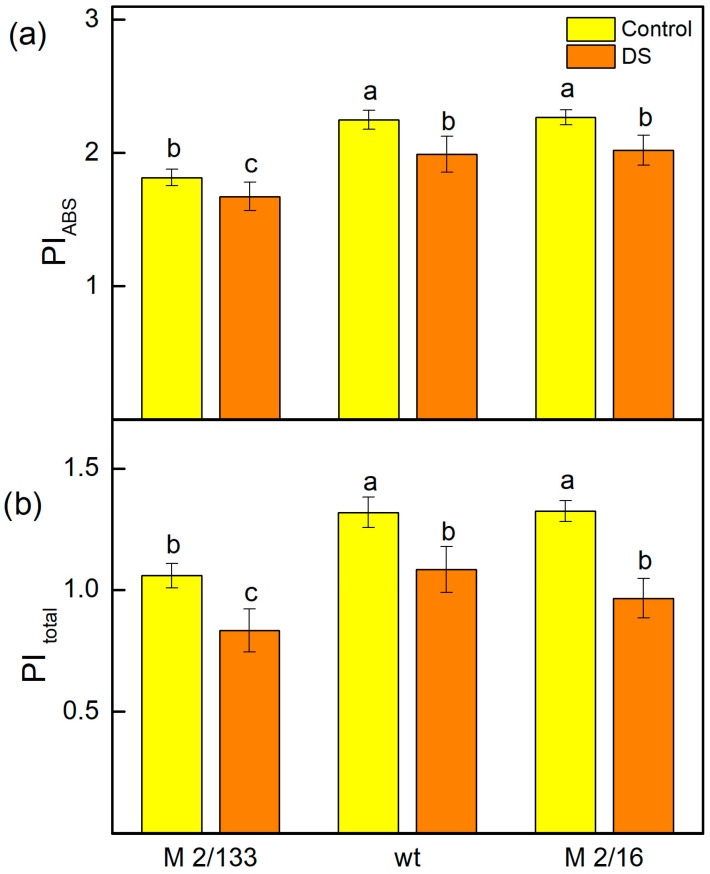
The performance indices PI_ABS_ (**a**) and PItotal (**b**) in Borec in wild type (wt) and its mutants *Costata* 2/133 (M 2/133) and *Coeruleovireus* 2/16 (M 2/16) in plants untreated (control) and treated with 20% PEG (DS). Different letters indicate significant differences among variants for respective parameters at *p* < 0.05.

The changes in the photosynthetic apparatus after drought stress led to a decrease in the performance indices (PI_ABS_ and PItotal) in all plants studied (Figure 9). The index PI_ABS_ was influenced by the active reaction centers per PSII antenna chlorophyll (parameter γRC/(1 − γRC)), the PSII primary photochemistry (φPo/(1 − φPo)), the thermal reactions of the intersystem electron carries (ψEo/(1 − ψEo)), and the PItotal was also influenced by the efficiency of the electron transport from Q_B_ to PSI acceptors (δREo/(1 − δREo)) [65,66,67]. The data also revealed that the values of both indices in plants with a higher number of LHCII oligomers (wt and M 2/16) after drought stress were similar to those of untreated plants of M 2/133, characterized by a smaller amount of LHCII oligomers. The differences in the PI_ABS_ and PItotal after drought stress were determined by the variation in the number of active reaction centers per PSII antenna chlorophyll (parameter γRC/(1 − γRC)) (Table 2). This parameter was higher in plants with a bigger degree of LHCII oligomerization, as it increased by 12% in wt and M 2/16 compared to M 2/133 (Figure 9).

### 2.6. Principal Component Analysis (PCA)

**Figure 10:**
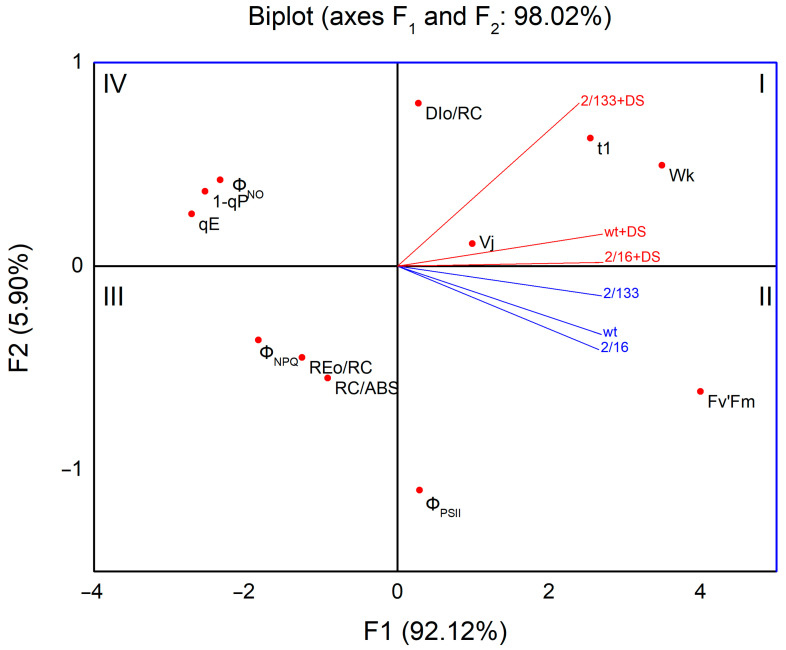
Principal component analysis showing variation among Borec wild type (wt) and its mutants *Costata* 2/133 (M 2/133) and *Coeruleovireus* 2/16 (M 2/16) in relation to selected parameters of chlorophyll a fluorescence in control and drought-stressed plants.

The first two principal components, F1 and F2, account for 98.02% of the total variability in the dataset, with the primary axis, F1, alone explaining 92.12% of the variance (Figure 10, Table S1). This indicates that the biplot provides an exceptionally accurate representation of the underlying physiological differences between the samples. The plants M 2/133, M 2/16 and wt under drought stress (DS), positioned on the right upper side of the F1 axis, exhibit a strong negative correlation with parameters related to photosynthetic efficiency and stability, specifically the density of active reaction centers (RC/ABS) and the electron transport flux per reaction center (REo/RC), which are located on the opposite side of the biplot. Simultaneously, a clear positive correlation is found in stressed samples with energy dissipation processes, such as dissipated energy per reaction center (DIo/RC), oxygen-evolving complex inactivation (Wk), and PSII acceptor side limitations (Vj). These factors influenced the Q_A_ reoxidation (t_1_). This cluster of parameters is located on the same side of the F1 axis, along with treated pea variants.

In contrast, the non-stressed plants, M 2/16, M 2/133, and the wild type, located on the right downside of the F1 axis, show a better photochemical performance. This is indicated by their strong positive correlation with key parameters of photosynthetic health and efficiency located in the same region, namely the maximum quantum yield of PSII at light-adapted state (Fv′/Fm′) and the effective quantum yield of energy conversion in PSII (ΦPSII). Accordingly, a negative relationship of control samples is observed with the fraction of closed RCs (1-qP), energy-dependent non-photochemical relaxation (qE), and unregulated energy losses from the PSII reaction center (Φ_NO_).

## 3. Discussion

The increasing frequency of droughts due to climate change poses a significant risk to plants and a serious challenge to maintaining food security [68]. Therefore, there is growing interest in studying photosynthetic tolerance as a tool to enhance plant production under adverse environmental conditions [69]. Photosynthesis is highly sensitive to abiotic stress factors, including water deficit. Previous investigations have revealed that the primary target of drought stress is the PSII complex in the photosynthetic apparatus [39,41], although the effects of this stress vary among different plant species [10,70]. The present study focuses, for the first time, on the role of the degree of LHCII oligomerization in determining plant sensitivity to drought stress.

It is known that water deficit causes oxidative damage in plant cells by inducing enhanced formation of ROS [68]. ROS can damage the photosynthetic apparatus, particularly PSII [71], due to inhibition of PSII repair and disruption of photosynthetic redox signaling pathways [72]. Data obtained in the current study revealed that H_2_O_2_ levels increased under drought stress only in plants with a lower degree of LHCII oligomerization (M 2/133 and wt) (Figure 1). This increase in H_2_O_2_ content corresponds with elevated levels of MDA, indicating lipid peroxidation in the thylakoid membranes of these plants (Figure 1), which is a result of oxidative damage to the membranes [73]. Our data suggests that under drought stress, oxidative damage is more pronounced in thylakoid membranes with a lower degree of LHCII oligomerization compared to those with higher oligomerization, leading to disruption of the membrane integrity and decreased membrane stability (MSI) (Figure 3). The variation in H_2_O_2_ and MDA levels observed among the studied plant species likely reflects differences in the regulation of antioxidant systems and stress-responsive transcription factors. Notably, MbICE1 overexpression in *Arabidopsis thaliana* has been shown to enhance stress tolerance by increasing chlorophyll and proline content, while reducing oxidative markers such as MDA, H_2_O_2_, and O_2_^−^ [24]. Similarly, MbWRKY50 promotes antioxidant enzyme activity, including superoxide dismutase and peroxidase, contributing to reduced ROS accumulation [23]. These findings align with our results, suggesting that transcriptional regulation plays a pivotal role in modulating oxidative stress responses. Although the role of VhMYB2 in the studied species remains to be fully elucidated, its potential involvement in regulating H_2_O_2_ and MDA levels cannot be excluded [25]. 

Additionally, numerous studies have shown that water deficit negatively affects photosynthetic pigments [74]. Chlorophylls *a* and *b* are the main antenna pigments that absorb light energy in LHCII and transfer it to the reaction centers of PSII, where charge separation occurs and further electron transport is initiated [75]. Under drought stress, enhanced degradation of Chl *a* has been registered in barley [76,77] and *Vigna radiata* [78]. It has also been shown that some drought-tolerant plant species preserve their leaf chlorophyll content under water deficit [74]. Considering these statements and the observed decrease in chlorophyll and carotenoid content only in the leaves of M 2/133 under drought stress (Figure 2), it can be assumed that pea plants with a higher degree of oligomerization are more tolerant of drought stress. Bearing in mind that carotenoids play an important role in the photoprotection [79,80] and the stabilization of the light-harvesting complexes of the photosynthetic apparatus [81,82], it can be supposed that this is one of the reasons for a greater resistance of plants with higher LHCII oligomerization. Furthermore, the protection of photosynthetic pigments under drought stress (Figure 2) also corresponds with a greater number of reaction centers per PSII antenna chlorophyll (RC/ABS) in plants with a higher degree of LHCII oligomerization (Figure 8a).

It has also been proposed that drought stress causes physical destabilization of the PSII core and some PSII reorganizations [43]. Giardi et al. [43] revealed a decreased amount of D1 protein due to its enhanced degradation. The study by Chen et al. [37] has shown a rapid disassembly of the LHCII under drought stress. These changes, which were most pronounced in M 2/133 may lead to a reduction in PSII efficiency (parameter Fv/Fm) under water deficit (Figure 4). A previous study on *Vicia faba* L. revealed that abiotic stress factors (cold and heat stress) significantly decrease Fv/Fm values depending on genotype tolerance [83]. Therefore, the maximal quantum efficiency of PSII (Fv/Fm) could be successfully used as an indicator of plant photosynthetic performance and plant tolerance under stress conditions [84,85]. The data in this study showed that the decrease in the Fv/Fm under drought stress corresponds with inhibition of the photosynthesis rate (parameter R_Fd_), which was more pronounced in M 2/133 (Figure 4c). 

Modifications in the donor and acceptor side of the LHCII–PSII complex in the studied plants [60] could alter their responses to drought stress (Figure 4 and Figure 5). Data from the present study revealed drought-induced changes in both the donor (Wk) and acceptor (Vj) sides of PSII (Figure 8). The increase in Wk, corresponding to inactivation of the OEC [86], was more pronounced in M 2/133. Moreover, considering that the Fv/Fo ratio is also related to the efficiency of OEC on the donor side of PSII [87], the current results also suggest stronger inhibition of oxygen-evolving activity in M 2/133 under drought stress conditions compared to the other studied plants (Figure 4b). The parameter Vj increased under drought stress, corresponding to changes in the PSII acceptor side, which influences the Q_A_ reoxidation (Table 1) and PSII functions. Our data revealed the decrease in the effective quantum yield of energy conversion (Φ_PSII_) under water deficit due to a higher fraction/amount of closed PSII reaction centers (1-qP), which is more enhanced in M 2/133, resulting from drought-induced changes in both the donor (Wk) and acceptor side of PSII (Vj) (Figure 8). Recently, Hu et al. [41] showed that parameter Φ_PSII_ is very sensitive to water deficiency and can be used for early detection of drought stress. The increase in the parameter 1-qP, along with the decrease in PSII efficiency (Φ_PSII_) and electron transport rate (ETR), was smaller under LL compared to that under HL (Figure 5). This suggests that drought-induced changes in PSII reduce its stability under high actinic light conditions. 

The present study also shows that the size of the PQ pool (N) decreased in all studied plants under drought stress, impacting the electron flux, reducing end electron acceptors at the PSI acceptor side (REo/RC) and the photosynthetic electron flow (ETR) (Figure 5c and Figure 8c). Kinetics of dark relaxation of chlorophyll fluorescence excited by a single saturating light pulse revealed an effect on Q_A_^−^ reoxidation under water deficit (Table 1). The fluorescence signal was fitted with two components: A_1_ (fast) and A_2_ (slow), with corresponding times t_1_ and t_2_, respectively, characterizing two pathways for Q_A_^−^ reoxidation [60,63,64,88,89]. A_1_ with time t_1_ is associated with interaction with PQ, while A_2_ with time t_2_ reflects the interaction with OEC (recombination of electrons on Q_A_Q_B_^−^ via the Q_A_^−^Q_B_↔ Q_A_Q_B_^−^). Data revealed that both time t_1_ and t_2_ increased after drought stress (Table 1), suggesting a delay of the Q_A_ reoxidation. The effect of drought stress on this process was more pronounced in membranes with lower LHCII oligomerization (M 2/133). Moreover, in these membranes, interaction with the OEC predominated, as indicated by a higher A_2_/A_1_ ratio in M 2/133 compared to the wild type and M 2/16. Similar effects on Q_A_^−^ reoxidation under salt stress have been reported in other plant species [89,90,91,92,93].

Furthermore, the dissipated energy flux per reaction center (DIo/RC) increased under drought stress in all studied plants (Figure 8b). Additionally, both non-regulated (Φ_NO_) and regulated energy losses (Φ_NPQ_) were enhanced after drought stress under both LL and HL actinic light (Figure 6). Under HL, the increase in the Φ_NPQ_ was greater than that of Φ_NO_. The increase in non-regulated losses (Φ_NO_) was significantly higher in M 2/133 under drought stress at both LL and HL actinic light compared to the wt and M 2/16. Our previous study showed modifications in the OEC in these mutant pea plants, which influenced the kinetic parameters of oxygen evolution and the ratio of active PSIIα to PSIIβ centers [59]. The proposed structural differences in the OEC may influence non-radiative charge recombination between P680+ and Q_A_^−^ [94]. Considering that non-regulated energy loss occurs within the PSII reaction center when Q_A_ is reduced [95], it has been suggested that Q_A_ reduction is a major requirement for efficient PSII reaction center-driven quenching [96]. The results of this study showed that higher non-regulated energy losses, which correlate with a more reduced Q_A_ state (Figure 5 and Figure 6), are associated with the generation of singlet excited oxygen under drought stress and greater damage in M 2/133. It is known that the non-photochemical quenching plays a significant role in regulating photosynthetic efficiency [97]. Determining the quantum yields of different components of non-photochemical quenching processes (ΦqE, ΦqT, ΦqI) provides more information about the dissipative mechanisms in the plants studied under drought stress (Figure 7). Data revealed that water deficit led to an increase in the quantum yield of energy-dependent quenching (ΦqE). The values of this component were similar in all studied plants. An additional rise in ΦqE was observed in all studied plants under drought stress at high actinic light (HL), in accordance with the previous statement that this component protects PSII from high-light intensity fluctuations [98]. State-transition quenching (ΦqT), which corresponds to the redistribution of excitation energy between the two photosystems, is vital for protecting the photosynthetic apparatus [99,100]. Experimental results showed that this parameter is significantly bigger in membranes with higher LHCII oligomerization, suggesting that this mechanism contributes to the enhanced protection of the photosynthetic apparatus in these plants under water deficit conditions. Furthermore, the quantum yield of photoinhibitory quenching (ΦqI) under drought stress, under LL and HL, increased more strongly in M 2/133, suggesting a bigger inhibition of the PSII function. The impact of drought stress on the ΦqI was stronger under HL than under LL (Figure 7), which corresponds with stronger inhibition of PSII function under HL in all studied plants.

Performance indexes reflect the functionality of both PSI and PSII and can give quantitative information on the current state of plant performance under stress [101]. Performance index (PI_ABS_) characterizes plant vitality [102] and includes the density of fully active reaction centers, electron transport efficiency, and the probability of photon capture, and gives information about the photosynthetic apparatus. Our data demonstrated that drought-induced changes in the photosynthetic apparatus also affected both performance indices PI_ABS_ and PItotal (Figure 9). A previous study revealed that the number of active reaction centers per PSII antenna chlorophyll is a major factor contributing to improved PSII efficiency (PI_ABS_) and overall photosynthetic performance (PItotal) in membranes with a higher degree of LHCII oligomerization [60]. Our experimental results now reveal that drought stress had a smaller influence on PI_ABS_ and PItotal in wt and M 2/16, i.e., in membranes with a higher LHCIIo/LHCIIm ratio. Our current results reveal that the values of PI_ABS_ and PItotal were bigger in membranes with a higher LHCIIo/LHCIIm ratio (wt and M 2/16). In addition, changes in the parameter γRC/(1 − γRC) suggest that better photosynthetic performance in plants with higher LHCII oligomerization results from better protection of the number of active reaction centers per PSII antenna chlorophyll (Table 2). The parameters φPo/(1 − φPo), ψEo/(1 − ψEo) and δREo/(1 − δREo) were similar across all studied plants (Table 2).

## 4. Materials and Methods

### 4.1. Plant Materials and Treatments 

The object of this study was *Pisum sativum* L. cv. Borec (wt) and its mutants *Costata* 2/133 (M 2/133) and *Coeruleovireus* 2/16 (M 2/16). The seeds of the wild type and mutants were kindly provided by the Institute of Plant Physiology and Genetics—Bulgarian Academy of Sciences. Plants were grown in 1/2 Hoaglands’ nutrient medium, which contains: 2.5 mM KNO_3_, 2.5 mM Ca(NO_3_)_2_, 1 mM MgSO_4_, 0.5 mM NH_4_NO_3_, 0.5 mM K_2_HPO_4_, 23 µM H_3_BO_3_, 4.5 µM MnCl_2_, 0.4 µM ZnSO_4_, 0.2 µM CuSO_4_, 0.25 µM Na_2_MoO_4_, and 20 µM Fe-EDTA [103]. Every three days, the nutrient solution was replaced. Plants were cultivated under controlled greenhouse conditions in the Institute of Biophysics and Biomedical Engineering—Bulgarian Academy of Sciences: 12 h photoperiod, day/night temperatures of 25/20 °C, a light intensity of 150 µmol/m^2^·s, and 70% relative humidity. After 14 days, the plants were subjected to drought stress (DS) by adding 20% polyethylene glycol (PEG 6000) to the nutrient solution [104]. After 3 days of drought stress, the corresponding measurements on fully developed leaves were made. Three independent experiments were carried out, each including four plants for variants.

### 4.2. Determination of Oxidative Stress Markers 

The procedure for the determination of H_2_O_2_ and MDA content in pea leaves is described in Yotsova et al. [105]. The leaf material (100 mg) was homogenized in 0.1% (*w*/*v*) trichloroacetic acid (TCA, 3 mL) on ice and then centrifuged at 14,000× *g* for 15 min at 4 °C. For the determination of the MDA, 1 mL of the clear supernatant was mixed with 1 mL of 20% TCA containing 0.5% thiobarbituric acid. After boiling for 25 min and subsequent cooling, the absorbance was recorded spectrophotometrically at 532 nm. For the determination of H_2_O_2_, 0.5 mL of the supernatant was mixed with 10 mM potassium iodide, and absorbance was measured at 390 nm. The amounts of H_2_O_2_ and MDA were calculated as in [105] and expressed on a dry weight basis (DW).4.3. Determination of Membrane Stability Index and Relative Water Content.

Membrane stability index (MSI) of pea leaves was measured as in the following equation: MSI (%) = [1 − (EC1/EC2)] × 100, where EC1 and EC2 are the conductivities of the leaf sample solution measured before (after incubation for 24 h) and after heating at 100 °C for 20 min. Leaf relative water content (RWC) was determined using the equation: RWC (%) = [(FW − DW)/(SW − DW)] × 100, where FW is leaf fresh weight, DW is leaf dry weight, and SW is saturated leaf weight [106].

### 4.3. Determination of Pigment Composition 

Photosynthetic pigments were extracted from leaf material, which was ground with ice-cold 80% acetone (*v*/*v*) in dim light. The suspension was centrifuged at 4500× *g* for 10 min at 4 °C. The supernatant was measured spectrophotometrically (UV-VIS Specord 210 Plus, Analytic Jena, Jena, Germany) to determine the content of the following pigments: total chlorophylls (Chl *a* and Chl *b*) and carotenoids (Car) according to the equation of Lichtenthaler [107], as described in Stefanov et al. [89]. Four independent replicates were measured for each variant and treatment. The pigment content was calculated and given as mg per g DW.

### 4.4. Pulse Amplitude Modulated Chlorophyll Fluorescence Measurements 

Pulse Amplitude Modulated (PAM) Chl *a* fluorescence was measured on dark-adapted leaf disks using a PAM fluorometer (Model 101/103, Heinz Walz GmbH, Effeltrich, Germany). For details, see Stefanov et al. [89]. The minimal fluorescence (F_0_) was measured at weak modulated light (0.02 µmol photons/m^2^·s). The maximum fluorescence in dark-adapted (Fm) and light-adapted (Fm′) states was induced by 3000 µmol photons/m^2^·s. Actinic light was 150 µmol photons/m^2^·s (LL) or 500 µmol photons/m^2^·s (HL). Parameters for characterization of the functions of the photosynthetic apparatus were as follows: the maximum quantum efficiency of PSII in the dark-adapted state(Fv/Fm); the ratio of quantum yields of photochemical to concurrent non-photochemical processes in PSII (Fv/Fo); the effective quantum yield of energy conversion in PSII (Φ_PSII_) [54,55]; PSII excitation pressure (1-qP) [108]; linear electron transport rate (ETR); nonregulated and regulated energy loss in PSII (Φ_NO_ and Φ_NPQ_); and the effective quantum yield of PSII photochemistry (Fv′/Fm′) [109,110]. In addition, components of non-photochemical quenching (the quantum yield of the energy-dependent quenching, ΦqE; the quantum yield of the state transition, ΦqT, and the quantum yield of the photoinhibitory quenching, ΦqI) were determined [111]. Parameter R_Fd_, corresponding to the rate of photosynthesis, was determined as in [62]. Time constants (t_1_, t_2_) and amplitude ratio (A_2_/A_1_) of the decay of variable Chl *a* relaxation after a saturating light pulse were also calculated (Table S2) [93]. 

### 4.5. Fast Chlorophyll a Fluorescence Measurements

Fast chlorophyll *a* fluorescence transients (OJIP curves) were recorded using a portable fluorometer (Handy PEA) on dark-adapted leaves (15 min). Measurements were conducted under ambient temperature and saturating light intensity (3000 μmol photons/m^2^·s). The following parameters were calculated using the JIP-test protocol, which evaluates the functionality of PSII and the overall photosynthetic apparatus: RC/ABS (density of active reaction centers per PSII antenna chlorophyll); REo/RC (electron flux reducing end electron acceptors at the PSI acceptor side per reaction center); Dio/RC (dissipated energy per reaction center); N (maximum turnovers of reduction Q_A_ until Fm was reached); Vj (relative variable fluorescence at the J-step); and Wk (the ratio of the K phase to the J phase). Performance index on absorption basis (PI_ABS_) and total performance index (PItotal) were also determined (Table S2):

PI_ABS_ = γRC/(1 − γRC)⋅ϕPo/(1 − ϕPo)⋅ψEo/(1 − ψEo)

PI_total_ = PI_ABS_⋅δRo/(1 − δRo)

All parameters were calculated using PEA plus software (version 1.13) provided with the fluorometer, following the standard JIP-test equations as described by Strasser et al. [102]. Measurements were replicated across 20 leaves per treatment group to ensure statistical robustness.

### 4.6. Statistical Analysis 

The mean values (±SE) were calculated from three independent treatments with three replicates for each variant. An ANOVA was performed, and the significance of the model was determined using the F-test. Following a significant F-test, differences between individual variants were identified using Tukey’s post hoc test, with values of *p* < 0.05 considered statistically significant. Distinct letters were used to denote statistically different groups. The dataset met the assumption of homogeneity of variances. Principal Component Analysis (PCA), a powerful multivariate statistical method, was employed to reduce the dimensionality of the extensive dataset and highlight its most influential variables [112]. This method also facilitated the evaluation of drought-induced structural and functional changes on pea variants differing in the LHCII oligomerization— Borec (wt) and two mutants, *Costata* 2/133 and *Coeruleovireus* 2/16, based on fluorescence parameters obtained from JIP-test and PAM measurements. All multivariate calculations included in the PCA cluster analysis [113], as well as data visualization, were generated with OriginPro 9 software (OriginLab Corporation, Northampton, MA, USA). 

## 5. Conclusions

This study highlights the important role of LHCII oligomerization in modulating plant responses to drought stress. Pea plants with a higher degree of LHCII oligomerization (M 2/16) demonstrated greater tolerance to water deficit, as evidenced by reduced lipid peroxidation and oxidative damage, preserved photosynthetic pigments, lower reduction in relative water content, and enhanced membrane stability. Under drought stress, these plants undergo smaller drought-induced changes in both donor and acceptor sides of PSII, accompanied by a greater number of active reaction centers per PSII antenna chlorophylls, higher PSII efficiency (Fv/Fm), electron transport rate (ETR), photosynthetic rate (R_Fd_), and sustained photosynthetic performance indices (PI_ABS_ and PItotal). At the same time, these plants exhibited more efficient energy dissipation mechanisms to prevent photodamage, including regulated and state transition quenching (Φ_NPQ_ and ΦqT). In contrast, plants with a lower LHCII oligomerization (M 2/133) showed increased levels of H_2_O_2_ and MDA, indicating elevated oxidative stress and lipid peroxidation. These plants also experienced greater disruption in PSII functions (Φ_PSII_, closed PSII centers), including delayed Q_A_ reoxidation and increased non-regulated energy losses (Φ_NO_), which correlated with a more reduced Q_A_ state, as well as inactivation of OEC (Wk). Therefore, the present study strongly demonstrates that the degree of LHCII oligomerization is a key factor in the determination of drought tolerance, influencing both membrane stability and functional efficiency of the photosynthetic apparatus. These findings provide new insights into the photoprotective strategies of plants and suggest that enhancing LHCII oligomerization could be a promising tool for improving crop tolerance to water deficit. Understanding plant defense mechanisms against drought stress is crucial for developing stress-tolerant crops in plant breeding processes and for ensuring global crop availability.

## Figures and Tables

**Table 1:** Kinetic characteristics of the dark relaxation of chlorophyll fluorescence induced by a single saturating light pulse in Borec wt and its mutants *Costata* 2/133 (M 2/133) and *Coeruleovireus* 2/16 (M 2/16): t_1_—time of the fast component; t_2_—time of the slow component; A_2_/A_1_—the ratio of the slow and fast components. The different letters indicate significant differences among variants for respective parameters at *p* < 0.05.

Variants	t_1_ (s)	t_2_ (s)	A_2_/A_1_
Control			
M 2/133	0.577 ± 0.045 ^b^	13.054 ± 0.732 ^c^	0.236 ± 0.012 ^b^
wt	0.626 ± 0.054 ^b^	16.046 ± 0.946 ^b^	0.206 ± 0.010 ^c^
M 2/16	0.621 ± 0.065 ^b^	16.811 ± 0.952 ^b^	0.205 ± 0.011 ^c^
+20% PEG			
M 2/133	0.732 ± 0.058 ^a^	18.870 ± 1.229 ^a^	0.301 ± 0.024 ^a^
wt	0.707 ± 0.043 ^a^	18.132 ± 1.229 ^a^	0.218 ± 0.013 ^c^
M 2/16	0.702 ± 0.045 ^a^	18.865 ± 1.235 ^a^	0.216 ± 0.011 ^c^

**Table 2:** Components of the performance indices PI_ABS_ and PItotal in Borec wild type (wt) and its mutants *Costata* 2/133 (M 2/133) and *Coeruleovireus* 2/16 (M 2/16) after treatment with 20% PEG. Significant differences between studied plants were determined by Student’s *t*-test and are indicated by asterisks at *p* ˂ 0.05 (*).

Variant	γRC/(1 − γRC)	φPo/(1 − φPo)	ψEo/(1 − ψEo)	δREo/(1 − δREo)
M 2/133	0.354 ± 0.007	5.005 ± 0.094	0.946 ± 0.062	0.494 ± 0.032
wt	0.398 ± 0.008 *	5.086 ± 0.207	0.981 ± 0.051	0.511 ± 0.027
M 2/16	0.399 ± 0.005 *	5.018 ± 0.155	1.005 ± 0.034	0.475 ± 0.023

## Data Availability

The original contributions presented in this study are included in the article/supplementary material. Further inquiries can be directed to the corresponding author.

## Supplementary Materials

The supporting information can be downloaded at: https://www.mdpi.com/article/10.3390/ijms262211078/s1.

## Abbreviations

The following abbreviations are used in this manuscript:

M 2/133	*Costata* 2/133
M 2/16	*Coeruleovireus* 2/16
DS	Drought stress
HL	High actinic light
H_2_O_2_	Hydrogen peroxide
PSI	Photosystem I
PSII	Photosystem II
Chl	Chlorophyll
Car	Carotenoids
LHCII	Light-harvesting complex of PSII
LL	Low actinic light
LHCIIm	Monomeric form of LHCII
LHCIIo	Oligomeric form of LHCII
OEC	oxygen-evolving complex
MDA	Malonaldehyde
MSI	Membrane stability index
wt	*Pisum sativum* L. Borec
RWC	Relative water content
