# Supplementary material for: The Role of the Organization of Light-Harvesting Complex II in the Drought Sensitivity of Pisum sativum L
Source: Int J Mol Sci. 2025 Nov 16;26(22):11078. doi: 10.3390/ijms262211078 (PMC12652389; doi:10.3390/ijms262211078)
Supplement: Supplementary file 1 [file ijms-26-11078-s001.zip › ijms-3943702-supplementary.pdf]

Role of the Organization of Light-harvesting Complex II for the Drought Sensitivity of *Pisum sativum* L.

Georgi D. Rashkov, Martin A. Stefanov, Preslava B. Borisova, Anelia G. Dobrikova and Emilia L. Apostolova*

Institute of Biophysics and Biomedical Engineering, Bulgarian Academy of Sciences, Acad. G. Bonchev Str., Bl. 21, Sofia, Bulgaria

Table S1. Variable loadings contributing to the principal component analysis model presented in Figure 10.

Parameters	F1	F2
Fv'Fm	4.00	-0.62
1-qP	-2.54	0.37
Φ_{PSII}	0.29	-1.10
Φ_{NO}	-2.34	0.42
Φ_{NPQ}	-1.83	-0.36
qE	-2.71	0.26
t1	2.55	0.63
RC/ABS	-0.92	-0.55
DIo/RC	0.28	0.80
REo/RC	-1.26	-0.45
Vj	0.99	0.11
Wk	3.49	0.50

Table S2. Description of the selected parameters of chlorophyll a fluorescence. All parameters are in relative units.

Fv/Fm	Maximum quantum efficiency of primary photochemistry of PSII [76,77]
Fv/Fo	Ratio of quantum yields of photochemical to concurrent non-photochemical processes in PSII [76,77]
R_{Fd}	The fluorescence decrease from Fm to a steady state chlorophyll fluorescence after continuous saturated illumination [62]
Fv′/Fm′	Effective quantum yield of PSII photochemistry [109,110]
1-qP	PSII excitation pressure [108]
ETR	Linear electron transport rate [109,110]
Φ_{PSII}	The effective quantum yield of energy conversion in PSII [76,77]
Φ_{NO}	Nonregulated energy loss in PSII [109,110]
Φ_{NPQ}	Regulated energy loss in PSII [109,110]
Φ_{qE}	Quantum yield of energy-dependent quenching [111]
Φ_{qT}	Quantum yield of state transition [111]
Φ_{qI}	Quantum yield of photoinhibition [111]
t_1 t_2	Time constants of the decay of variable Chl *a* relaxation after a saturating light pulse [93]
A_2/A_1	Amplitude ratio of the decay of variable Chl *a* relaxation after a saturating light pulse [93]
JIP parameters [102]	
RC/ABS	Number of active PSII reaction centers per absorbed photon, indicating the structural integrity of PSII units
DIo/RC	Amount of energy lost as heat or fluorescence per active reaction center
REo/RC	Efficiency of electron transfer from Q_A^- to PSI end acceptors per active reaction center
Vj	Accumulation of Q_A^- and the efficiency of electron transport from PSII to Q_A
N	Number of times Q_A is reduced during the measurement period (until reaching Fm), relative size of the plastoquinone pool, which is involved in electron transport whitie photosynthetic apparatus
Wk	Ratio between J phase and K phase
PI_{ABS}	Parameter reflecting the overall photosynthetic performance, integrating energy conservation from photons absorbed by PSII to the reduction of intersystem electron acceptors
PItotal	Extends PI_{ABS} by including the efficiency of electron transport beyond PSII to PSI end acceptors, offering a more comprehensive view of the photosynthetic electron transport chain.
